# Complications of Immediate versus Delayed DIEP Reconstruction: A Meta-Analysis of Comparative Studies

**DOI:** 10.3390/cancers14174272

**Published:** 2022-09-01

**Authors:** André S. Alves, Vincent Tan, Matteo Scampa, Daniel F. Kalbermatten, Carlo M. Oranges

**Affiliations:** Department of Plastic, Reconstructive, and Aesthetic Surgery, Geneva University Hospitals, Geneva University, 1205 Geneva, Switzerland

**Keywords:** DIEP, autologous reconstruction, free flap, immediate breast reconstruction, delayed breast reconstruction, adverse events, surgical timing, radiotherapy

## Abstract

**Simple Summary:**

Although the deep inferior epigastric perforator flap (DIEP) has become the most frequent autologous flap in breast reconstruction, it remains unclear whether reconstruction should be performed at the same time as the mastectomy or delayed. Therefore, we conducted a meta-analysis to offer an overview of recipient site postoperative complications and help guide practicians toward the ideal timing for breast reconstruction. A pooled analysis using the Mantel and Haenszel methods with a fixed effect model provided results as an odd ratio with a 95% confidence interval. Among most complications including hematoma, infection, fat necrosis, and flap loss, no significant differences were observed. However, delayed wound healing was significantly higher for patients who underwent delayed breast reconstruction. This paper offers evidence that both surgical timings offer similar outcomes and are, therefore, valid surgical strategies.

**Abstract:**

Purpose: The setting regarding the ideal timing for deep inferior epigastric perforator flap (DIEP) reconstruction remains unclear. Immediate breast reconstruction (IBR) is performed at the same time as mastectomy, while delayed breast reconstruction (DBR) is performed at any time after mastectomy except immediately. We compared both strategies to assess whether IBR or DBR should be performed to reduce postoperative adverse events. Methods: A systematic review of PubMed, Embase, Medline, Cochrane, and Web of Science was conducted, aiming at articles comparing the recipient site outcomes of IBR versus DBR with DIEP. We used the Mantel–Haenszel method with a fixed effects model. Results were expressed as the OR with a 95% CI. Results: Two retrospective and two prospective studies were identified involving 5784 DIEPs (1744 immediate and 4040 delayed). We showed a significant difference in favor of IBR for wound healing issues (OR = 0.57, 95% CI 0.41, 0.77; *p* = 0.0003). However, no significant differences for hematoma, infection, fat necrosis, partial flap loss, and total flap loss rate were seen. Conclusions: Despite variability in the choice of the ideal time for breast reconstruction and outcomes reported among studies, immediate DIEP surgery appears to be a reliable setting with less delayed healing issues.

## 1. Introduction

First used in 1989 [1] and then popularized in 1994 [2], the deep inferior epigastric perforator flap (DIEP) has become one of the most popular techniques for breast reconstruction [3]. This autologous procedure has many advantages compared to implant-based reconstruction such as no prothesis, no capsular contracture, a natural aesthetic shape, and a higher satisfaction rate [4]. Compared to other autologous flaps such as the transversus rectus abdominis muscle flap (TRAM) or the latissimus dorsi flap (LD), the DIEP carries many qualities including low donor site morbidity (fat necrosis, abdominal wall hernia) and less postoperative pain, at the price of longer operation time and microsurgical skill requirement [5,6,7]. Common postoperative complications such as abdominal bulging, wound dehiscence, seroma, infection, and hematoma have been reported either on the recipient site or on the donor site [8,9,10]. 

**Figure 1:**
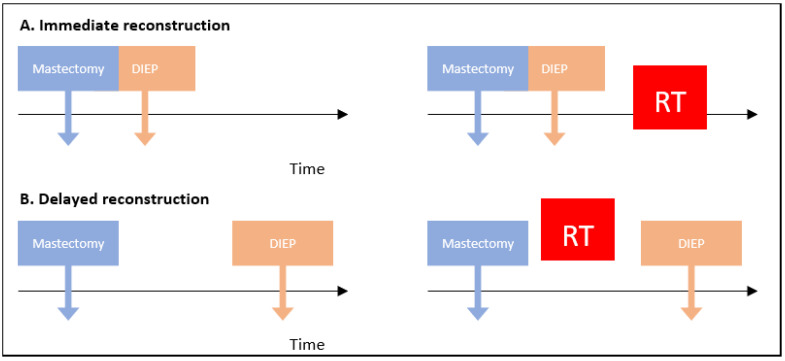
Time options for breast reconstruction. RT = radiotherapy; DIEP = deep inferior epigastric perforator flap.

However, little attention has been focused on comparing complications in immediate versus delayed reconstruction. Two breast reconstruction strategies are possible after mastectomy, and the choice of the best setting depends on multiple factors. The first, immediate breast reconstruction (IBR), is performed at the same time as mastectomy, while the second, delayed breast reconstruction (DBR), is performed at any time after mastectomy except immediately (Figure 1). Delayed immediate reconstruction is also one strategy for breast reconstruction. It consists of tissue expander insertion before definitive breast reconstruction, sometimes preferred when adjuvant radiotherapy is necessary [11]. Because this strategy involves in most cases a distant definitive reconstruction, it was classified in the DBR group in the selected articles. IBR is known to have reduced recovery time, less psychological distress, and greater cost-effectiveness compared to DBR [12,13,14]. The last option is preferentially used in settings where adjuvant therapies such as radiation or chemotherapy are indicated, due to their association with potential flap complications (flap shrinkage and inconvenient scar formation) and morbidity rate in IBR [15]. Therefore, in daily practice, plastic surgeons frequently choose DBR for patients requiring adjuvant treatment. 

Although breast reconstruction techniques have been widely investigated, there is a lack of evidence regarding how the appropriate timing for surgery affects postoperative adverse events. Therefore, the aim of this meta-analysis was to evaluate the quality and strengths of the current evidence regarding surgical complications on the recipient site between IBR and DBR with DIEP flaps. Both strategies were assessed to know whether IBR or DBR should be used to reduce postoperative adverse events. 

## 2. Materials and Methods

We performed a meta-analysis of comparative studies in accordance with the PRISMA 2020 guidelines for reporting meta-analyses [16]. This meta-analysis was registered on Research Registry, ID: reviewregistry1437. 

### 2.1. Search Strategy 

A systematic review was conducted on 3 June 2022 using the following databases: PubMed, Embase, Medline, Cochrane, and Web of Science. Language was restricted to English. The keywords “immediate”, “delayed”, and “DIEP” were used as search strings. 

### 2.2. Article Selection

All articles comparing the postoperative outcomes on the recipient site between immediate and delayed DIEP reconstruction after a mastectomy were selected for a qualitative analysis. No limitations were applied on the age of the patients or their ethnicity. Review articles, case reports, conference abstracts, simulation studies, and clinical studies in nonhuman subjects were not included. Studies involving patients who received other types of autologous reconstruction, implant-based reconstructions, or papers with no postoperative outcome measures were also removed. We decided to exclude studies where the overall population underwent radiotherapy to have a representation as close as possible to reality with the heterogenous group (Table 1). 

Two authors (A.S.A. and V.T.) independently identified the relevant studies on the basis of the title and the abstract. Selected articles were then fully read. If they met all selection criteria, data were extracted independently by the two authors. In case of disagreement, it was solved after consultation with the senior author (C.M.O.). 

### 2.3. Data Extraction 

The following variables were extracted: the name of the study, the study design, the total number of DIEPs, the number of IBRs, the number of DBRs, and the minor and major complications. Two authors (A.S.A. and V.T.) independently identified these parameters, and disagreements were resolved after discussion with the senior author (C.M.O.). 

### 2.4. Outcome of Interest 

The mean interest of our study was to evaluate minor and major complications after IBR and DBR with the DIEP procedure. As minor complications, we selected wound healing issues (healing delayed, dehiscence, and superficial skin necrosis), hematoma, infection, and fat necrosis, while major complications were limited to partial or total flap loss. 

### 2.5. Statistical Analysis

When two or more studies reported outcome data, these were pooled using Review Manager 5.4.1 software (The Cochrane Collaboration, The Nordic Cochrane Center, Copenhagen, Denmark). Odds ratios with 95% CI were used to evaluate dichotomous outcomes (reconstruction complications). Rates of each complication ((1) wound healing issues, (2) hematoma, (3) infection, (4) fat necrosis, and (5) partial or total flap loss) were compared for IBR and DBR. 

Before performing the synthesized analysis, heterogeneity between studies was assessed in Review Manager 5.4.1 using the Higgins and Thompson I^2^ statistics. Levels of heterogeneity were defined as low and high heterogeneity if I^2^ < 50% and I^2^ ≥ 50%, respectively. In cases of low heterogeneity, we used the fixed effect Mantel–Haenszel model [17]. In the case of high heterogeneity, we applied the random effect DerSimonian and Laird model [18] assuming that part of the high heterogeneity was independent of fixe variables. Results of meta-analyses are shown as forest plots. Funnel plots were used to check the risk of publication bias. All statistical tests were two-sided, and statistical significance was defined as *p* < 0.05.

## 3. Results

### 3.1. Search Result

**Figure 2:**
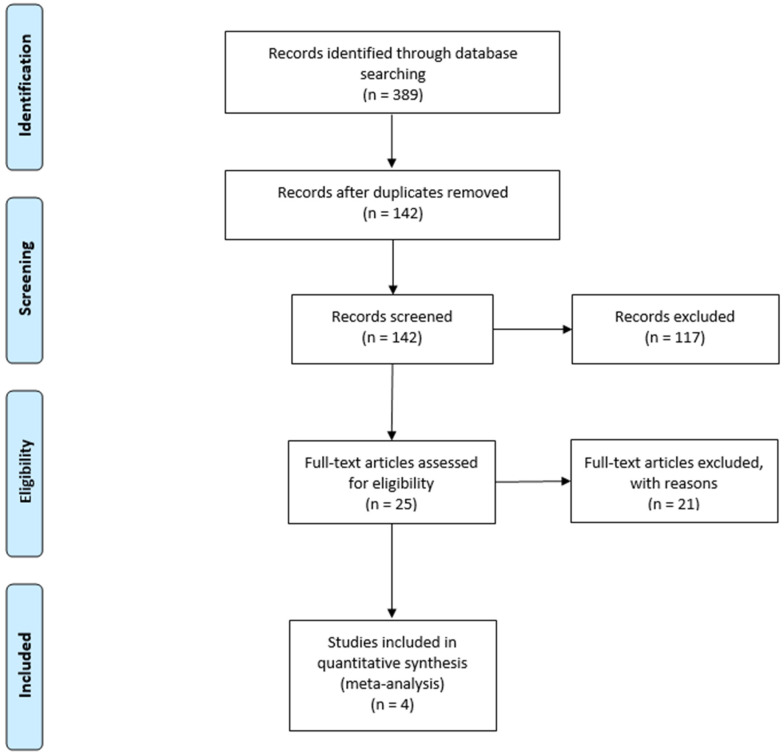
Flow diagram of search for eligible studies.

A total of 389 studies were identified. After deduplications and review of the title and abstract, 25 articles were selected for full-text review. Of these, two retrospective and two prospective studies met all the selection criteria and were analyzed [8,9,14,19] (Figure 2). 

**Figure 3:**
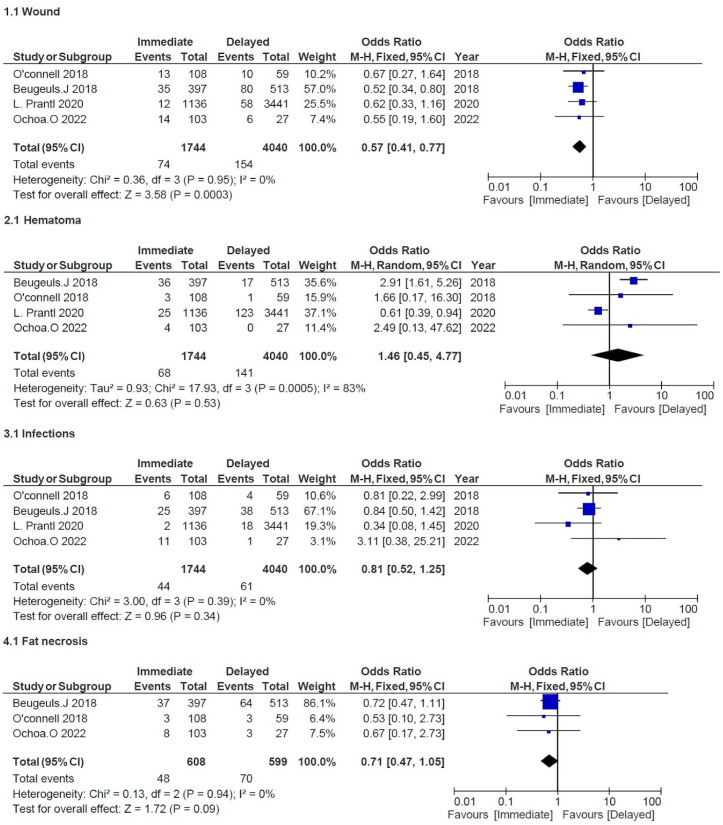
Combined ORs to assess effect of immediate versus delayed DIEP on adverse events for minor complication: (**1.1**) wound healing, (**2.1**) hematoma, (**3.1**) infections, and (**4.1**) fat necrosis [8,9,14,19]. Blue shapes correspond to individual studies. Squares size is proportional to the weight of the study while black diamonds shapes correspond to pooled studies.

**Figure 4:**
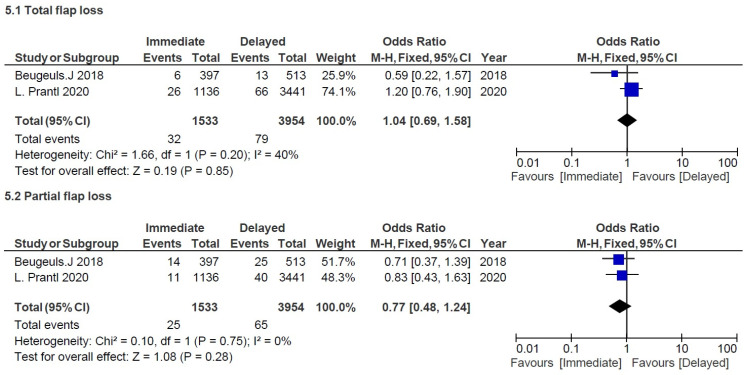
Combined ORs to assess effect of immediate versus delayed DIEP on adverse events for major complications: (**5.1**) total flop loss, and (**5.2**) partial flap loss [8,9]. Blue shapes correspond to individual studies. Squares size is proportional to the weight of the study while black diamonds shapes correspond to pooled studies.

The four studies mentioned above were aimed at comparing several recipient site outcomes following DBR and IBR using the DIEP technique. Prantl et al. and Beugels et al. were both multicentric and national (Germany and the Netherlands) studies while Ochoa et al. and O’Connell et al. were monocentric. The meta-analysis covered a total of 5784 DIEPs, of which 1744 were immediate and 4040 were delayed [8,9,14,19] (Table 2). All studies were recent and covered a similar data collection period after 2009. Some adverse events differed in these studies but were always compared between IBR and DBR. All outcomes were reported if they happened after a follow-up period except for Prantl et al., which collected data on adverse events only if revision surgery was required. However, we considered that it was still relevant to include the latter study because the proportions of complications between both groups remained the same whether they required surgical revision or not. Our hypothesis proved to be relevant since our studies were comparable with an I^2^ equal or close to 0 for each outcome except for hematoma (Figure 3 and Figure 4). All studies except O’Connell et al. included unilateral and bilateral flap procedures. Population age was homogeneous across papers with adult women around the age of 50. 

### 3.2. Minor Complications

This meta-analysis showed with all articles a significant difference in favor of IBR for wound healing (OR = 0.57, 95% CI 0.41, 0.77; *p* = 0.0003) but not for hematoma (OR = 1.46, 95% CI 0.45, 4.77; *p* = 0.53) or infection (OR = 0.81, 95% CI 0.52, 1.25; *p* = 0.34) [8,9,14,19]. Three studies reported fat necrosis, and no significant difference was found (OR = 0.71, 95% CI 0.47, 1.05; *p* = 0.09) [8,14,19]. Wound healing issues were defined in all studies as healing disturbances predisposing patients to delayed healing. For this outcome, Beugels et al. included patients with wound dehiscence and superficial skin necrosis. Ochoa et al. classified patients with wound problems if they required dressing changes, debridement, or vacuum-assisted or operative debridement. O’Connell et al. documented women with wound problems if the wound was not fully healed in 30 days or more after surgery. Lastly, Prantl et al. collected data of patients with wound-healing disturbances only if they required revision surgery. Concerning hematoma or infections, no description was made among studies except in Ochoa et al. where infection was classified depending on if patients required oral antibiotics, intravenous antibiotics, or surgical debridement. Three studies documented fat necrosis among other outcomes. Beugels et al. defined this outcome as a palpable firmness detected by physical examination or ultrasound. O’Connell et al. documented fat necrosis when excision surgery was required. 

### 3.3. Major Complications

No significant differences were observed for partial flap loss (OR = 0.77, 95% CI 0.48, 1.24; *p* = 0.28) and total flap loss (OR = 1.04, 95% CI 0.69, 1.58; *p* = 0.85). Beugels et al. and Prantl et al. documented these last outcomes as a major complication requiring re-exploration for some patients. 

### 3.4. Further Analysis

**Figure 5:**
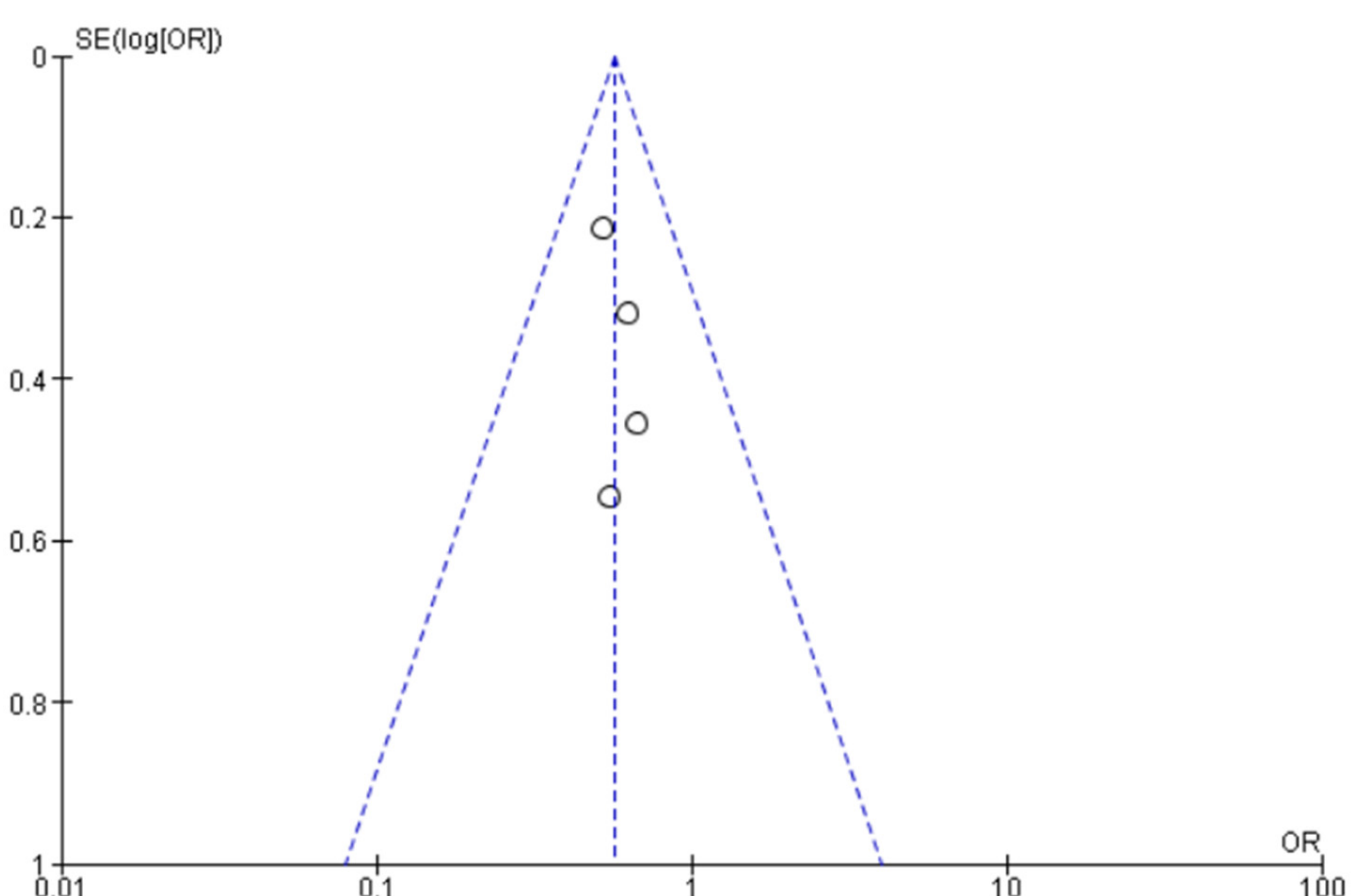
Funnel plot of comparison of adverse events for wound.

A funnel plot was established to assess possible publication bias for every subgroup. The standard error of log (OR) of each study was plotted against its log (OR), and no bias was found (Figure 5). 

## 4. Discussion

The present meta-analysis, based on two retrospective and two prospective studies, reviewed all existing evidence on recipient site postoperative outcomes following IBR versus DBR with DIEP flap. This is the first meta-analysis of postoperative recipient site outcomes to our knowledge. No statistically significant difference was found in terms of hematoma, infection, fat necrosis, and partial or total flap loss. However, wound healing issues (healing delayed, dehiscence, and superficial skin necrosis) showed a significantly higher rate in DBR. Our findings suggest that adverse events do not seem to occur more frequently according to surgical timing except for delayed healing (wound healing issues). 

The use of radiotherapy or chemotherapy as adjunctive oncological therapies could possibly explain some adverse events seen among DBR. Some patients with infiltrative breast cancer such as T3 to T4 with or without lymph node involvement are more likely to undergo radiotherapy before performing breast reconstruction [20,21,22]. Most patients requiring radiotherapy were found in the DBR group in relation to traditional guidelines. A few studies showed that DBR was preferable compared to IBR when multimodal treatment was required to reduce complications and increase aesthetic result [15,19,23]. Among other benefits, it has the advantage of replacing tissues damaged from radiation. In a delayed postradiotherapy setting, resection of irradiated skin between the mastectomy scar and the inframammary layer is commonly realized [24]. Even if DBR is preferably chosen in this case, wound healing problems such as wound dehiscence and superficial skin necrosis are still more frequent in DBR compared to IBR and could be due to radiation exposure. A sub-analysis of the association between surgical wound healing and the use of radiation therapy could help identify how the later affected cicatrization. However, radiation therapy was inconsistently reported in selected studies, not allowing this additional analysis. One article showed higher wound healing issues in IBR compared to DBR when patients received PMRT (17.2% vs. 7.6%, *p* < 0.01) [25]. In addition, surgical site infection might be favored by irradiation, but we only showed a trend in favor of IBR without statistical significance [26,27]. However, recent meta-analyses, one including patients with adjuvant radiotherapy and the other including all type of flaps, demonstrated no optimal evidence in terms of overall complications for immediate versus delayed reconstruction after postmastectomy radiotherapy [25,28]. 

As opposed to radiotherapy, chemotherapeutic agents such as tamoxifen are rather implicated in flap complications due to thrombotic effects that could potentially lead to flap loss [29,30]. Oncologic drugs could, therefore, affect surgical outcome in breast reconstructions. Even if it was not significant in our analysis, we showed a trend in favor of IBR concerning partial flap loss. This finding supports the fact that chemotherapy, which is more prevalent in DBR, may have an impact on the abovementioned outcome. Interestingly, some papers showed that, when IBR was performed even after neoadjuvant chemotherapy, no morbidity increase was found as opposed to DBR [31,32]. The need for oncologic treatment should, therefore, not be an obstacle to an IBR, as suggested by previous practice. 

Mastectomy followed by breast reconstruction presents intraoperative circumstances that could increase hematoma development. Until now, no contributing factor has been clearly attributed to this outcome [33]. However, Seth et al. (2013) reported a preferential location of hematomas originating at the level of the pectoralis muscle and the axillary region. In our study, a trend was found in favor of delayed reconstruction, but it was not significant. This trend could perhaps be explained by the fact that IBR required generally extensive procedure with axillary node dissection during mastectomy, which increased the risk of active bleeding and hematoma formation [8]. Despite no risk factors being clearly associated with this outcome, perioperative measures could be introduced to reduce hematoma incidence. Plastic surgeons could prevent this outcome intraoperatively with intravenous tranexamic acid that has been associated with reduced risk of hematoma without increasing thromboembolic event [34]. Postoperatively, meticulous attention should be paid to the axilla region and surgical drains to monitor a bleed into the chest wall musculature. 

The DIEP flap preparation could have a major impact on fat necrosis. A systematic review reported four principal factors increasing this outcome: perforator perfusion zones, perforator location, perforator number, and venous congestion [35]. They concluded that a minimum of two to three perforators of a sufficient caliber should be used, and Holm zones 3 and 4 should be spared to reduce fat necrosis. Computed tomographic angiography has become the current preoperative tool to evaluate vascular variability and quality [36]. Indocyanine green angiography provides intraoperative information to surgeons regarding tissue perfusion and the prevention of flap necrosis [37]. Our results did not show any significance regarding this adverse event but a trend in favor of IBR. A hypothesis could be due to radiotherapy which was more frequent in DBR than IBR, and which could lead to vessel damage and promote necrosis [38]. A study in a rat model demonstrated that ischemic preconditioning of the recipient site with deferoxamine could prevent flap graft necrosis by increasing angiogenesis, capillary neoformation, and vascular growth factor protein expression [39]. 

One of the most important aspects to discuss with patients is the aesthetic result with both techniques to reduce the psychological impact of the cancer. BREAST-Q is a valid scale that has been used into daily practice to evaluate the quality of life after breast surgery [40]. Unfortunately, selected articles did not report aesthetic satisfaction scores or reported them in a manner impeding meta-analysis of this outcome. Consequently, we could not include this variable in our analysis. Even though this aspect represents a limitation of the study, we found some articles discussing aesthetic outcome. Results showed that IBR is a better strategy in term of aesthetic outcome following 45 months after reconstructions [41]. For many patients, skin/nipple-sparing mastectomy and IBR result in a breast where it is difficult to tell there ever was a mastectomy [42]. However, similar satisfaction rates have previously been reported among patients undergoing IBR or DBR [14,43]. It seems that, in both settings, overall satisfaction with breast reconstruction improved over time since the procedure [44,45]. In situations where the patient will require PMRT, IBR with autologous tissue flaps can have quite problematic long-term outcomes. Therefore, DBR should be considered to not interfere with aesthetic outcomes. Minimizing the emotional impact and the best preservation of the natural aspect of the breast should be essential. In addition to complications, patient satisfaction should be considered in the balance. 

Concerning the economic aspect, it is clearly stated that IBR is more cost-effective compared to DBR. An efficient healthcare system is a priority for any university hospital. It is known that cost-effectiveness is a key issue in the choice of medical treatments. A study compared the total cost between IBR and DBR with the DIEP procedure. This research highlighted a significant difference between both strategies with a financial cost of almost 35% less for IBR compared to DBR [12]. Delayed surgery was the costliest mostly due to the additional anesthetic drug, the costs of surgical materials, and the administrative paperwork. Another study showed no difference, but it also did not consider the abovementioned parameters that made the difference in the final cost [46]. 

The limitations of this meta-analysis are attributed to the small number of included studies. This is mostly explained by this topic only having recently been investigated. However, we believe that the quality of the selected studies offsets the number of papers. Moreover, it allowed comprehensive knowledge and statistical analysis of all available data to provide guidance in breast surgery reconstruction. All studies were published in the same period, and investigated outcomes presented a low heterogeneity (I^2^ = 0). Selected articles were placed toward the top of the funnel plot, indicating that they were large studies with high power. Moreover, our funnel plot was symmetrical, indicating a low probability of having highly heterogeneous results and differences in methodological quality [47]. We had large patient (*n* = 4904) and DIEP (*n* = 5784) samples. Lastly, the proportion of PMRT in the immediate (20%) and delayed (45%) groups was almost the same across studies. 

In our articles, oncological outcomes such as tumor recurrence and overall survival (OS) were not reported. A previous study showed an increased risk of recurrence (1.7%) in women receiving delayed DIEP flap reconstruction compared with those who received an immediate reconstruction [48]. Regarding OS, IBR was associated with higher survival compared to DBR [25]. 

## 5. Conclusions

This meta-analysis provides new knowledge regarding adverse events depending on the time of procedure. This study revealed a higher prevalence of wound healing issues (healing delayed, dehiscence, and superficial skin necrosis) in delayed breast reconstruction, probably linked to complex care processes where radiation or chemotherapy are more frequent and could interfere in this setting. In times of health system efficiency, immediate breast reconstruction is known to be the best choice among both strategies in terms of total cost. However, plastic surgeons should also be aware of complications that may preferentially appear in one or the other setting. A shared decision-making process with patients should be essential to provide better insight into several complications.

## Figures and Tables

**Table 1:** PICOS table features.

PICOS	Inclusion	Exclusion
Population	Adults who underwent breast reconstruction after mastectomy	Study where the overall population received radiotherapy
Intervention	Autologous breast reconstruction with DIEP flap	Other types of autologous reconstruction, implant-based reconstructions
Comparator	Reconstruction timing (immediate versus delayed)	
Outcomes	Recipient site complications	Studies that did not report recipient site complications
Study design	Comparative studies	Review articles, meta-analysis, case reports, conference abstracts, simulation studies, clinical studies in nonhuman subjects, and unpublished studies

**Table 2:** Characteristics of the included studies.

Author	Year	Study Period	N° Patients	Mean (SD) Age IBR	Mean (SD) Age DBR	Total of DIEP	Immediate (IBR)	Delayed (DBR)	PMRT before IBR	PMRT before DBR	Follow-Up
Beugels [8]	2018	2010–2017	737	50.7(9.4)	51.0(8.6)	910	397	513	21.7%	44.4%	9–10 m
O’Connell [19]	2018	2009–2014	167	§	§	167	108	59	25.9%	100.0%	§
Prantl [9]	2020	2011–2019	3927	49.9(11.5)	51.8(35.8)	4577	1136	3441	18.5%	41.6%	3 m
Ochoa [14]	2022	2012–2016	73	*	*	130	103	27	28.1%	50.0%	282–303 d

IBR = immediate breast reconstruction, DBR = delayed breast reconstruction, PMRT = post-mastectomy radiotherapy; * median age 52 (22–73) for IBR and 47.5 (41–61) for DBR; § data not reported for all IBR and DBR; m = months, d = days
